# Desiccation Induces Accumulations of Antheraxanthin and Zeaxanthin in Intertidal Macro-Alga *Ulva pertusa* (Chlorophyta)

**DOI:** 10.1371/journal.pone.0072929

**Published:** 2013-09-05

**Authors:** Xiujun Xie, Shan Gao, Wenhui Gu, Guanghua Pan, Guangce Wang

**Affiliations:** 1 Tianjin Key Laboratory of Marine Resources and Chemistry, College of Marine Science and Engineering, Tianjin University of Science and Technology, Tianjin, China; 2 Institute of Oceanology, Chinese Academy of Sciences, Qingdao, China; 3 Graduate School, Chinese Academy of Sciences, Beijing, China; Mount Allison University, Canada

## Abstract

For plants and algae, exposure to high light levels is deleterious to their photosynthetic machineries. It also can accelerate water evaporation and thus potentially lead to drought stress. Most photosynthetic organisms protect themselves against high light caused photodamages by xanthophyll cycle-dependent thermal energy dissipation. It is generally accepted that high light activates xanthophyll cycle. However, the relationship between xanthophyll cycle and drought stress remains ambiguous. Herein, *Ulva pertusa* (Chlorophyta), a representative perennial intertidal macro-algae species with high drought-tolerant capabilities and simple structures, was used to investigate the operation of xanthophyll cycle during desiccation in air. The results indicate that desiccation under dim light induced accumulation of antheraxanthin (Ax) and zeaxanthin (Zx) at the expense of violaxanthin (Vx). This accumulation could be arrested by dithiothreitol completely and by uncoupler (carbonyl cyanide p-trifluoromethoxyphenylhydrazone) partially, implying the participation of Vx de-epoxidase in conversion of Vx to Ax and Zx. Treatment with inhibitors of electron transport along thylakoid membrane, e.g. DCMU, PG and DBMIB, did not significantly arrest desiccation-induced accumulation of Ax and Zx. We propose that for *U. pertusa*, besides excess light, desiccation itself could also induce accumulation of Ax and Zx. This accumulation could proceed without electron transport along thylakoid membrane, and is possibly resulting from the reduction of thylakoid lumen volume during desiccation. Considering the pleiotropic effects of Ax and Zx, accumulated Ax and Zx may function in protecting thylakoid membrane and enhancing thermal quenching during emersion in air.

## Introduction

Light energy is necessary for plants and algae to conduct photosynthesis; however, it is also deleterious to the photosynthetic machineries when the absorbed light energy exceeds the plant’s photosynthetic capacities (e.g., under high light or/and drought conditions) [1], [2], [3], [4]. Plants prevent high light-induced photodamage through various mechanisms, including the xanthophyll cycle (Xc) and antioxidant systems [5], [6], [7], [8]. Exposure to high light levels also can accelerate water evaporation, thus placing plants at risk of a water deficit. Because exposure to high light levels can lead to both photodamage and a water deficit, it is possible that the regulatory mechanisms used by plants or algae to deal with these problems may be correlated. Most of the existing model systems based on higher plants are not appropriate for studying this potential correlation because they have evolved sophisticated organs to prevent severe water loss (e.g., stomata and cuticle), and very few of them can recover from disastrous drought stress. A suitable model system for examining inherent correlations should be morphologically as simple as possible and also possess strong desiccation-tolerant capabilities.

Intertidal macro-algae (e.g., *Porphyra* (Rhodophyta) and *Ulva* (Chlorophyta)) are among the most important resurrection plants. They are composed of only one or two layers of cells, lack sophisticated tissue differentiation, and frequently and periodically experience extreme abiotic stresses [9], [10]. For example, *Porphyra* sp. and *Ulva* sp. often experience severe (80–95%) water loss during low tide, and the thalli can temporarily turn into crisp sheets [11], [12], [13], [14]. Thalli emersed from seawater also may be exposed to direct sunlight (e.g., at noon on a sunny day), which can have deleterious effects on photosynthetic apparatus and thylakoid membrane [9], [15]. However, after rehydration these algae can recover from the detrimental effects of desiccation. Thus, intertidal macro-algae represent an optimal system for investigating the correlation between the mechanisms used by plants and algae to respond to high light and drought stress.

Most of intertidal macro-algae use a number of different strategies to cope with high intensity illumination [9], [10], [11]. The Xc is one of the most important mechanisms to cope with high light stress, in which accumulated zeaxanthin and antheraxanthin converted from violaxanthin facilitate the transition of light harvesting complex II from the state of light capture to that of quenching [16], [17], [18], [19]. Recently, Fernández-Marín *et al.* (2011) reported that over-night desiccation treatment in closed chamber of 75% relative humidity without illumination could induce significant accumulations of Ax and Zx in brown and green algae [20]. For most intertidal macro-algae, however, desiccation often develops in air within 3–6 hours depending on their positions, and sometimes is accompanied by direct exposure to sun light. To our knowledge, not much is known concerning the operation of Xc during desiccation in air, especially under high irradiance, which seems more important for intertidal macro-algae to survive during low tide in the middle of sunny day.

Herein, specimens of *Ulva pertusa*, a representative perennial intertidal macro-alga species composed of two layers of cells, were used to investigate the operation of Xc during desiccation in air under various light levels, and its dependence on the electron transport along thylakoid membrane.

## Results

### Severe Water Loss Induced Accumulations of Ax and Zx

**Figure 1:**
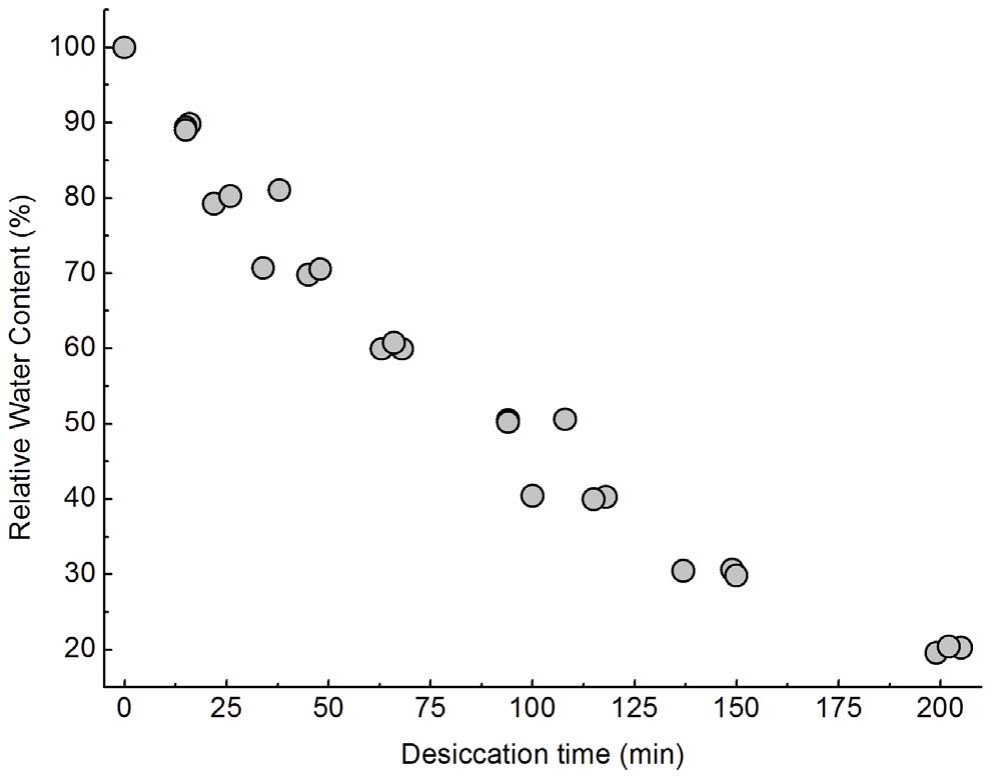
Relative water content in the thalli of *U. pertusa* desiccated in air. It took about 3.3 hours to dehydrate the thalli to relative water content of 20%. For the calculation of relative water content, see “Materials and Methods”. Experiments were performed under dim light at room temperature of about 20°C.

**Figure 2:**
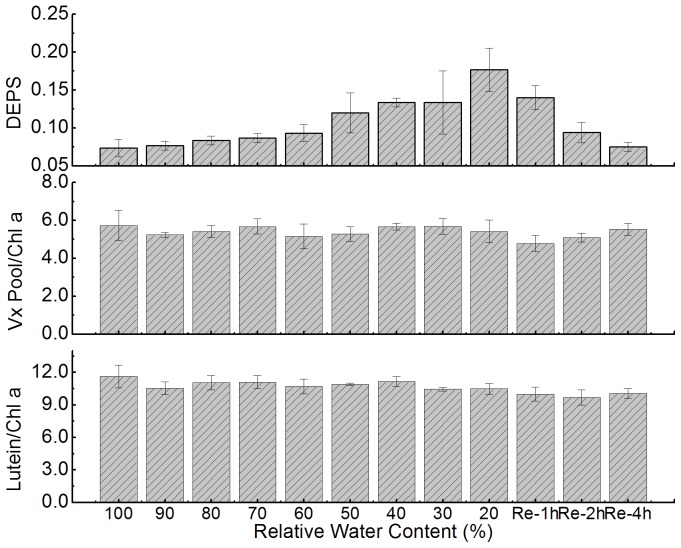
Effects of varying levels of desiccation on the xanthophyll cycle and lutein content of *U. pertusa* thalli. Thalli of *U. pertusa* were emersed in air under dim light (0.7 µmol m^–2^ s^–1^) for desiccation treatment and then rehydrated in seawater for 1, 2 and 4 hours. The xanthophyll cycle was activated by desiccation as indicated by the increase in the de-epoxidation state (DEPS) value, which is calculated as (Ax+Zx)/(Vx+Ax+Zx). The relative content of the Vx pool (Vx+Ax+Zx) and lutein is presented as µg per mg of chlorophyll a; this also applies to Figs. 3, 5 and 6. Error bars represent SD for n = 3.

The thalli of *U. pertusa* were subjected to desiccation in air under dim light (0.7 µmol m^−2^s^−1^). The desiccation dynamics is shown in Fig. 1; after about 3.3 hours of emersion in air, large amounts of water lost, leaving the thalli at about 20% relative water content. Desiccation induced significant accumulations of Ax and Zx in the thalli of *U. pertusa* (Fig. 2). The de-epoxidation state (DEPS), which was used to depict the relative content of Ax and Zx in Xc pigments pool and was calculated as (Ax +Zx)/(Vx +Ax +Zx), significantly increased after severe desiccation under dim light. For fully hydrated thalli, the DEPS was about 0.073±0.012, whereas it was 0.177±0.029 for desiccated thalli after ∼3.3 h emersion in air. After rehydration under darkness, DEPS started to decrease till four hours immersed under seawater, when the DEPS was 0.08±0.006, comparable to that for control (P>0.05).

The total pool size of Xc pigments, which reportedly influences the epoxidation state or DEPS [21], was not decreased during desiccation and rehydration. The amount of lutein, which plays great role in photoprotection in higher plants [22], did not decline during dessication and rehydration either.

### Effects of Light Intensities on the Operation of Xc in the Thalli of *U. pertusa* during Desiccation in Air

**Figure 3:**
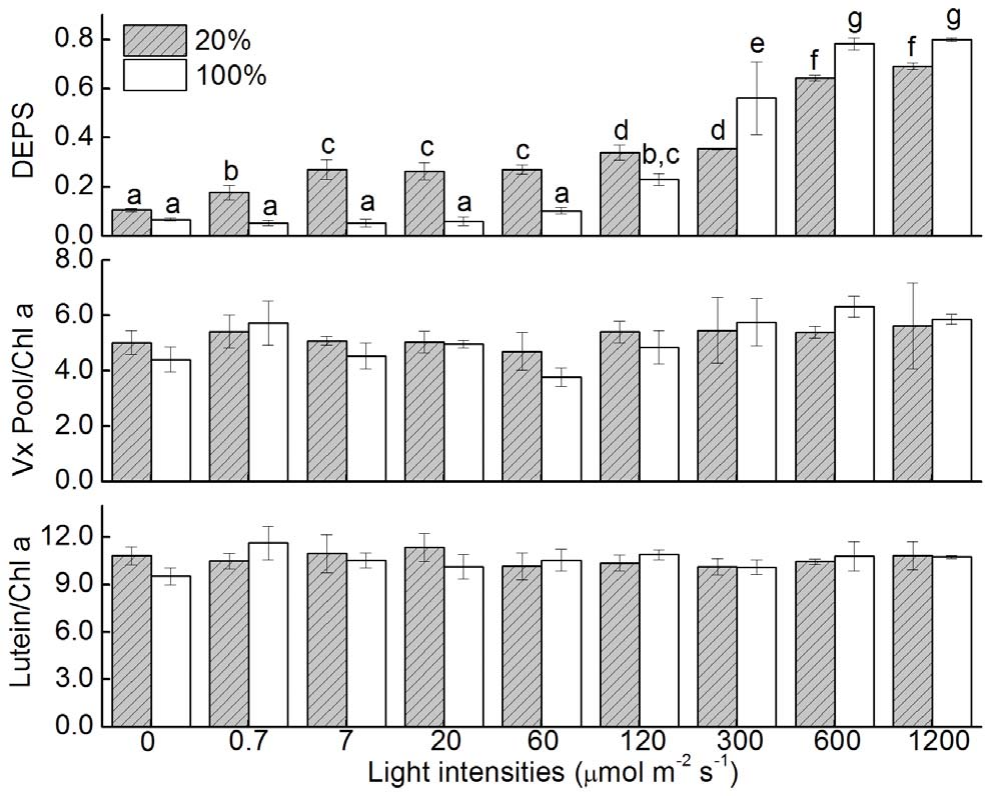
Responses of fully hydrated and emersed *U. pertusa* thalli to varying light intensities. For each light intensity tested, thalli of *U. pertusa* were emersed in air to desiccate to a water content of 20% or immersed in seawater (100%). Treatment times of desiccated and hydrated thalli were comparable. Error bars represent SD for n = 3.

We then examined the effects of various light intensities on the Xc of *U. pertusa* during desiccation in air (Fig. 3). The DEPS differed significantly between desiccated thalli (The thalli referred to as desiccated hereafter in the text had a water content of ∼20%, except where otherwise indicated) and fully hydrated thalli under low light conditions. The Xc in the thalli emersed in air was more susceptible to light compared to those immersed in seawater. At a light intensity of 7 µmol m^–2^ s^–1^, the DEPS for desiccated thalli was 0.27±0.04, significantly higher than that for hydrated thalli (0.053±0.015) (P<0.05). The Xc in the hydrated thalli clearly was not activated under these moderate light conditions (0–60 µmol m^−2^s^−1^). The light intensity required for hydrated thalli to activate the Xc was at least 120 µmol m^–2^ s^–1^,when the DEPS for hydrated thalli increased to 0.231±0.024, although it remained lower than that of desiccated thalli (0.339±0.031). When the light intensities were higher than 120 µmol m^−2^s^−1^, the DEPS was relative lower for the desiccated thalli compared to fully hydrated ones. The DEPS for both groups reached maximal under light intensity of 600 µmol m^−2^s^−1^: 0.64±0.01 for desiccated thalli and 0.78±0.02 for fully hydrated thalli.

**Figure 4:**
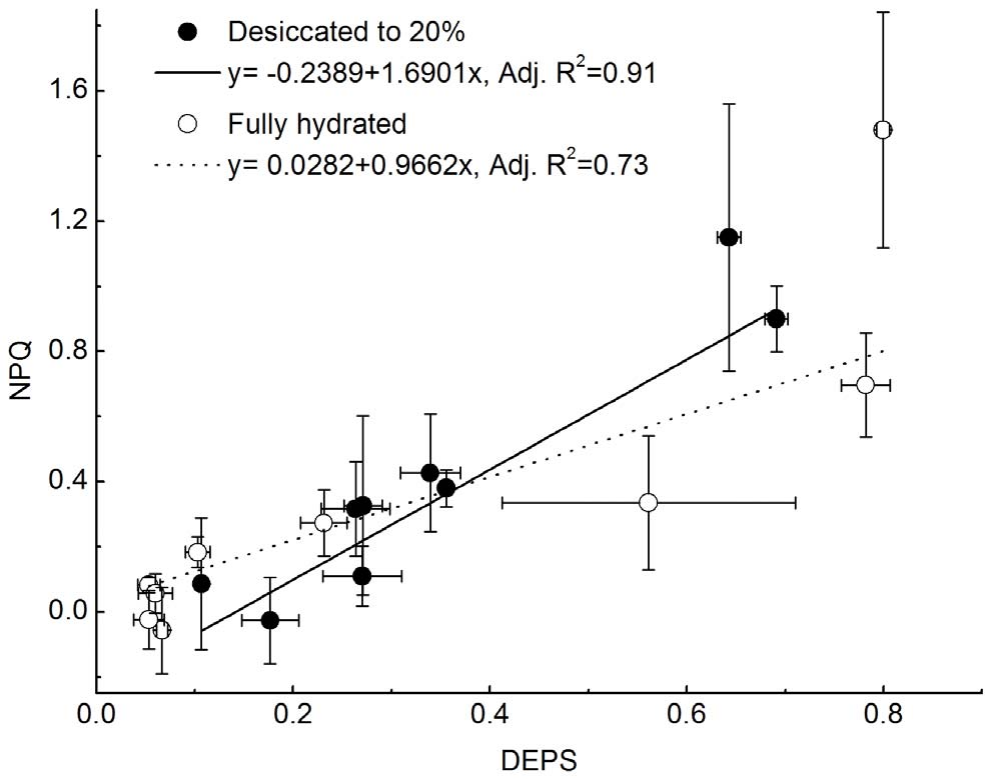
Linear correlations between non-photochemical quenching (NPQ) and the de-epoxidation state (DEPS) in desiccated and in fully hydrated thalli of *U. pertusa*. Experimental conditions were as in Fig. 3. Vertical and horizontal bars represents SD for n = 3.

The effects of desiccation on NPQ also were investigated. As is shown in Fig. 4, NPQ and DEPS exhibited a positive linear correlation in both desiccated and hydrated thalli. The results of covariance analysis showed that there is no significant difference between the desiccated group and fully hydrated group with respect to the responses of NPQ to DEPS (p>0.05).

### DTT Inhibited Desiccation-induced Accumulations of Ax and Zx

**Figure 5:**
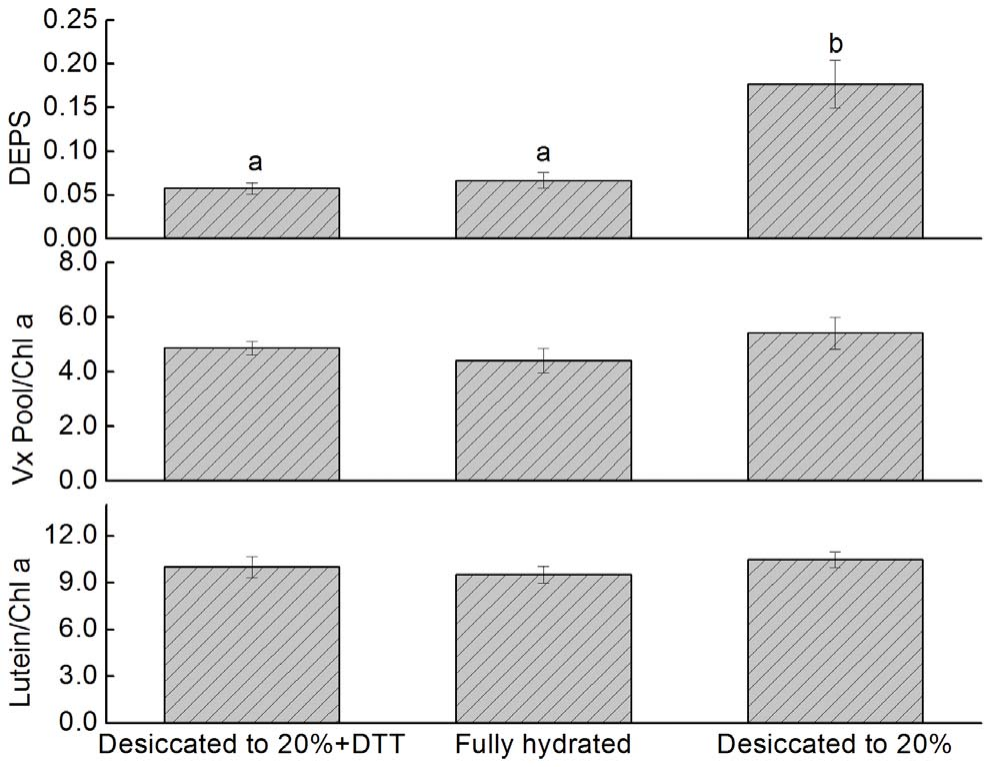
Effects of dithiothreitol (DTT) on the Xc and lutein content of *U. pertusa* thalli during desiccation in air under dim light. DTT was infiltrated into thalli before desiccation treatment. Experimental conditions were as in Fig. 2. Error bars represent SD for n = 3.

To confirm that the observed increases in the DEPS were due to activation of the enzyme violaxanthin de-epoxidase (VDE), dithiothreitol (DTT) was infiltrated into the thalli before desiccation treatment. DTT exerts inhibition effects by reducing the disulfide bonds of VDE [23]. The DEPS for the fully hydrated thalli (0.067±0.009) differed significantly (p<0.05) from that of desiccated thalli without DTT pretreatment (0.177±0.028) (Fig. 5). However, a desiccation-induced increase in the DEPS was not found in the DTT-pretreated thalli, for which the value was (0.058±0.006); this value was comparable to that of fully hydrated thalli (p>0.05). This result suggests that VDE catalyzed the conversion of Vx to Ax and Zx under severe desiccation in air.

### Effects of Metabolic Inhibitors on the Accumulation of Ax and Zx during Desiccation under Dim Environment

**Figure 6:**
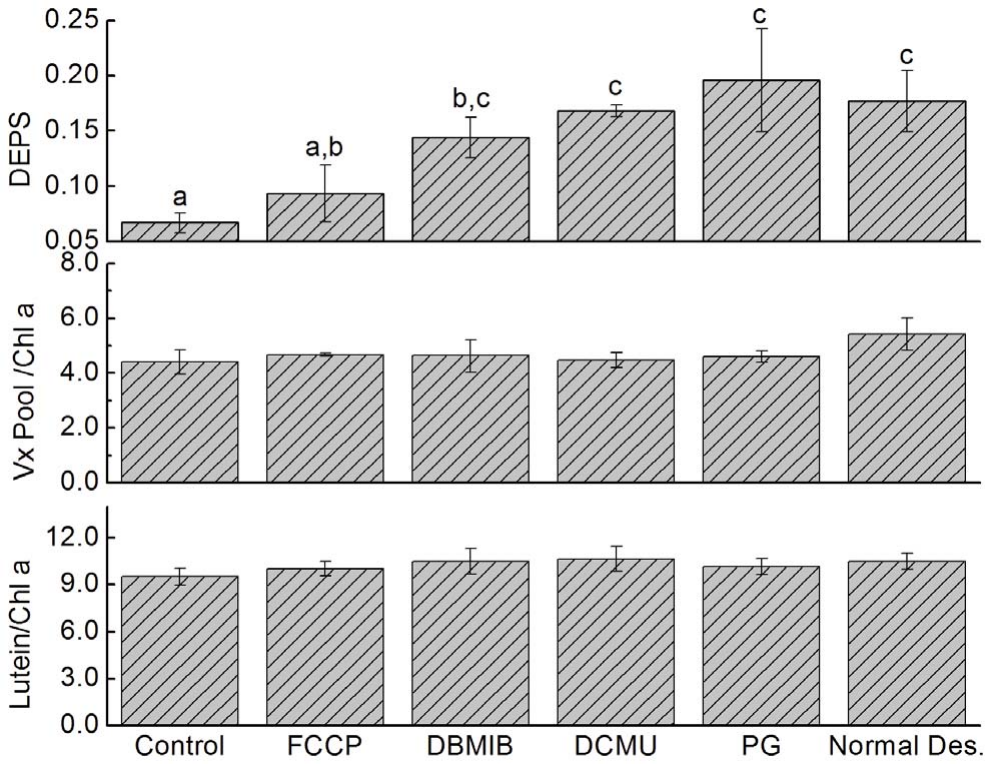
Effects of an uncoupler and various metabolic inhibitors on the Xc and lutein content of *U. pertusa* thalli during desiccation in air under dim light. Chemicals were infiltrated into thalli before desiccation treatment. The de-epoxidation state (DEPS) and the contents of the Vx pool and lutein were measured after severe water loss (∼20% water content), except in the control, which was fully hydrated. Normal Des. represents desiccation of thalli without any inhibitor treatment. Experimental conditions were as in Fig. 2. Error bars represent SD for n = 3.

It is well known that the activation of VDE requires acidification of the thylakoid lumen [24]. Carbonyl cyanide p-trifluoromethoxyphenylhydrazone (FCCP), which is an efficient uncoupler that dissipates the proton gradient across the thylakoid membrane, significantly attenuated the increase in the DEPS of desiccated thalli from 0.177±0.028 to 0.093±0.026 (Fig. 6). Given that the DEPS for hydrated thalli was 0.067±0.009, the inhibition percentage of FCCP, which was calculated as (DEPS_normal des._ – DEPS_inhibitor_)/(DEPS_normal des._− DEPS_control_)×100%, was 76%. This result implies that ΔpH plays a crucial role in the increase in the DEPS during desiccation.

In the chloroplast, the trans-thylakoid proton gradient is formed by vertical proton transport from stroma to lumen coupled with electron transport, including linear electron flow (LEF), chlororespiration, and cyclic electron flow around PSI (CEF1). To clarify which factor(s) affect activation of the Xc during desiccation, various specific inhibitors were infiltrated into the thalli before desiccation treatment, and the results are shown in Fig. 6. Propyl gallate (PG), which effectively inhibits chlororespiration by blocking the oxidation of plastoquinol catalyzed by PTOX [25] and 3-(3,4-dichlorophenyl)-1,1-dimethylurea (DCMU), which inhibits electron transports in PSII by binding to the Q_B_ sites in D1 protein, had a negligible effect on the desiccation-induced increase of the DEPS (p>0.05): the DEPS was 0.196±0.047 for PG-pretreated desiccated thalli and 0.193±0.076 for DCMU-pretreated desiccated thalli compared to 0.177±0.028 for inhibitor-free desiccated thalli. These results suggest that chlororespiration and LEF were not necessary for activation of the Xc induced by desiccation. Although the DEPS for the dibromothymoquinone (DBMIB, which blocks the electron flow at cytb6f complex, thus inhibits both LEF and CEF1)-pretreated desiccated thalli was 30% lower than the value for inhibitor-free desiccated thalli, there was no significant difference according to the statistical analysis (P>0.05).

## Discussion

### Desiccation Triggers the Accumulation of Ax and Zx at Low Light Levels

It is generally accepted that terrestrial plants evolved from the ocean via the coastal intertidal zones, wherein organisms endured periodic desiccation in air and potential direct exposure to high light levels. In particular, most macro-algae are sessile and therefore cannot circumvent stressors; instead they have to adapt to them. The adaptive mechanisms that have evolved in these algae to enable them to survive in these harsh environments remain to be determined.

The predominant problem encountered by intertidal macro-algae is severe water loss and concomitant salt stress during low tide [9], [11]. Moreover, if the low tide occurs during the day, emersion in air also can lead to high light stress. Numerous studies have shown that either water loss or high intensity illumination can cause oxidative damage and disruption of membrane [1], [2], [12], [26]. To acclimate these stress environment, higher plants [27], [28] and macro-algae [9], [12] have developed numerous mechanisms. Figs. 2 and 3 show that the Xc of the thalli of *U. pertusa* can be activated by exposure to high light levels (100% groups in Fig. 3) and by desiccation in air, even under very dim light (Fig. 2 and 20% groups in Fig. 3). De-epoxidized xanthophylls, e.g. Ax and Zx, have been demonstrated to exert pleiotropic effects, including as antioxidant to scavenge reactive oxygen species (ROS), preventing lipid peroxidation [19], [29], [30], [31], [32], and as allosteric modulator of qE, facilitating the formation of thermal energy quenching [16]. Meanwhile, it is also noteworthy that the capabilities to accumulate Ax and Zx is lower for desiccated thalli compared with fully hydrated ones under higher light intensities (i.e. when light intensities are 300, 600 and 1200 µmol m^−2^s^−1^ in Fig. 3), suggesting Xc alone may not be sufficient for *U. pertusa* to survive severe desiccation. Other mechanisms e.g. increase production of various antioxidants to diminish oxidative damages caused by desiccation [12], and rapid recovery of PSI activities after rehydration [13], [33], may also crucial for *Ulva* to survive. Finally, samples used in this work were collected during autumn, and thus had periodically experienced high light illumination and emersion during summer. These environmental factors affect many aspects, including the profile of photosynthetic pigments [27], [34]; thus are also important for their remarkable tolerance to stress.

### Desiccation Induced Accumulation of Ax and Zx could Proceed without Electron Transport along Thylakoid Membrane

The inhibitory effects of DTT and FCCP (Figs. 5 and 6) suggest that the desiccation-induced accumulation of Ax and Zx, as indicated by the increase of the DEPS, were attributable to activation of VDE by the acidification of the thylakoid lumen. This is consistent with experimental results reported in other publications [24], [35], [36]. In the chloroplast, acidification of the thylakoid lumen is always coupled with electron flow along a series of electron carriers, including chlororespiration, LEF, and CEF1. Although studies have shown that chlororespiration can trigger the Xc in diatoms, which accumulates diatoxanthin converted from diadinoxanthin, to dissipate excess absorbed energy [37], [38], our results show that the contribution of chlororespiration to the desiccation-induced conversion of Vx to Ax and Zx in *U. pertusa* was negligible (Fig. 6), which is consistent with traditional views on the different optimal pH of VDE and of diadinoxanthin de-epoxidase [17]. The null inhibitory effects of DCMU and DBMIB suggest that LEF and CEF1 were also not the main factor contributing to the buildup of ΔpH (Fig. 6). Thus, it seems that desiccation induced accumulations of Ax and Zx under low light was not correlated with electron transports along thylakoid membrane.

So, it’s possible that the structural changes of thylakoid membrane during desiccation induced the conversion of Vx to Zx. Cruz *et al.* (2001) reported that the thylakoid lumen in *Chlamydomonas reinhardtii* shrank significantly after hyperosmotic treatment [39]. It seems reasonable to assume that the decrease of the volume of thylakoid lumen would also occur in *U. pertusa* during desiccation since air-drying and immersion in hyperosmotic solution have comparable physiological influences [26]. Contraction of thylakoid lumen would lead to increase of the relative concentrations of proteins within lumen, like VDE. Thus, the interactions between the VDE and the thylakoid membrane become more frequent. Meanwhile, evaporation of water during desiccation in air results in increased proton concentration within the lumen since the thylakoid membrane is impermeable for protons. Although ATPase is present in thylakoid membrane functioning as proton transporter, its conductivity for protons would be considerably lessened during desiccation. All of these factors together may possibly contribute to the accumulation of Ax and Zx during desiccation in air under dim light. However, exact reasons for this phenomenon remain to be further investigated.

## Materials and Methods

Samples of *U. pertusa* were collected between October and December 2011 from the intertidal zone in Qingdao (36° 05′ N, 120° 18′ E), Shandong Province, China. This location is not privately-owned or protected in any way, thus no specific permissions were required, and the field studies did not involve endangered or protected species. Thalli were rinsed with sterile seawater to remove contaminants or epiphytes, and healthy thalli (i.e., those with a Fv/Fm value >0.8 measured by chlorophyll fluorescence with dual-PAM) were used for the following experiments.

### Desiccation Treatment

Thalli were desiccated in air at room temperature of (20°C). Relative water content (in %) was calculated as:
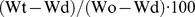
where Wt is the weight of thalli after desiccation for time t; Wd is the dry weight of the thalli dried at 80°C for 12 h; Wo is the original weight of the thalli after gentle removal of the surface water with tissue paper [13].

### Treatment with Metabolic Inhibitors

Thalli subjecting to desiccation were firstly treated with tissue paper to completely remove surface water and then immersed into seawater containing various inhibitors for 1 h under darkness. To evaluate the contributions of VDE and ΔpH to the increase of the DEPS during desiccation, 3 mM DTT [23] or 10 μΜ FCCP was infiltrated into the thalli before desiccation.

To assess the contributions of electron flow along the thylakoid membrane to the activation of VDE, thalli were treated with various metabolic inhibitors before desiccation. DCMU, which binds to the Q_B_ site on the D1 protein and thus blocks the electron supply to the photosynthetic electron chain from water, was used to treat thalli at a final concentration of 10 µM. DBMIB, which blocks electron flow from plastoquinol to the cyt b6/f complex, was applied at a final concentration of 80 µM [13].

### Pigment Extraction and Analysis

All pigment extraction procedures were performed under low temperature of 0°C and dim lighting,where light intensities were less than 2 µmol m^−2^s^−1^. Samples, which were preserved in liquid nitrogen after the desiccation treatments, were ground in a mortar with liquid nitrogen. Pigments were extracted with 5 ml of methanol: acetone (1∶1, v/v) per 200 mg wet weight of thalli for about 60 min. The extracts were centrifuged for 3 min at 10,000 g and the supernatants were filtered through a 0.22 µm syringe filter into HPLC vials for HPLC analysis.

Pigment separation and quantification were performed using an Agilent 1200 HPLC equipped with an Rx-C18 analytical column (4.6×250 mm) and Quatpump (Agilent Technologies Inc., Santa Clara, CA, USA). The separation method was modified from Thayer *et al.* (1990) [40]. The column were balanced with initial solvent consisting of water: methanol: acetonitrile: acetyl acetate (5∶30∶65∶0) for 10 min at flow rate of 0.8 ml/min. The pigments separation was started by a 5 min linear gradient from the initial solvent to water: methanol: acetonitrile: acetyl acetate (0∶15∶85∶0), and then isocratically continued for 7 min. Eluents then was transited to water: methanol: acetonitrile: acetyl acetate (0∶45∶35∶20) within 2 min, followed by 16 min linear gradient to water: methanol: acetonitrile: acetyl acetate (0∶45∶0∶55). The column oven temperature was set as 20°C. Chlorophyll a, lutein, and Zx standards were obtained from Sigma (St. Louis, MO, USA), and Vx and Ax were obtained from the International Laboratory USA (South San Francisco, CA, USA).

### Measurements of Chlorophyll Fluorescence

Chlorophyll fluorescence was measured using the Dual-PAM-100 system (Walz GmbH, Effeltrich, Germany). Non-photochemical quenching (NPQ) was calculated as: NPQ = (Fm – Fm’)/Fm’ [41], where Fm is the maximal fluorescence of the dark-adapted thalli before desiccation treatment induced by a saturation pulse (10,000 μ mol m^–2^ s^–1^) and Fm’ is the maximal fluorescence of the desiccated thalli with various relative water contents.

### Statistical Analysis

All the results were presented as mean values ± SD of three independent experiments. Statistical analyses were performed using the IBM SPSS Statistics 19 package (IBM Co., Armonk, New York, USA). One-way ANOVA and Duncan post-hoc test (α = 0.05) were used to determine whether significant differences were exist between various treatment groups. Covariance analysis was used to compare the difference between desiccated group and fully hydrated group in the response of NPQ to DEPS using treatment (fully hydrated and desiccated) as fixed factor and DEPS as covariate (ANCOVA). OriginPro 8.5.0 SR1 (OriginLab Co., Northampton, MA, USA) was used to perform linear fitting and to plot graphs.
